# Prevalence of gestational diabetes mellitus in Pakistan: a systematic review and meta-analysis

**DOI:** 10.1186/s12884-024-06290-9

**Published:** 2024-02-03

**Authors:** Muhammad Adnan, Muhammad Aasim

**Affiliations:** Health Research Institute, National Institute of Health, Islamabad, Pakistan

**Keywords:** Diabetes Gestational, Glucose Tolerance Test, Pakistan, Pregnancy, Prevalence

## Abstract

**Background:**

A variety of screening tools and criteria are used for the diagnosis of gestational diabetes mellitus (GDM). As a result, the prevalence rate of GDM varied from 4.41% to 57.90% among studies from Pakistan. Beside this disagreement, similar multi-centric studies, community surveys and pooled evidence were lacking from the country. Therefore, this first systematic review and meta-analysis aimed to measure the overall and subgroup pooled estimates of GDM and explore the methodological variations among studies for any inconsistency.

**Methods:**

Using the PRISMA guidelines, seventy studies were identified from PubMed, ScienceDirect, Google Scholar and PakMediNet database. Of them, twenty-four relevant studies were considered for systematic review and nine eligible studies selected for meta-analysis. AXIS was used for measuring quality of reporting, I^^2^ statistics for heterogeneity among studies and subgroups, funnel plot for reporting potential publication bias and forest plot for presenting pooled estimates.

**Results:**

The pooled sample of nine studies was 27,034 (126 – 12,450) pregnant women, of any gestational age, from all four provinces of Pakistan. Overall pooled estimate of GDM was 16.7% (95% CI 13.1 – 21.1). The highest subgroup pooled estimate of GDM observed in studies from Balochistan (35.8%), followed by Islamabad (23.9%), Khyber Pakhtunkhwa (17.2%), Sindh (13.2%), and Punjab (11.4%). The studies that adopted 75g 2-h OGTT had a little lower pooled estimate (16.3% vs. 17.3%); and that adopted diagnostic cut-off values [≥ 92 (F), ≥ 180 (1-h) and ≥ 153 (2-h)] had a greater pooled estimate (25.4% vs. 15.8%). The studies that adopted Carpenter criteria demonstrated the highest subgroup pooled estimate of GDM (26.3%), after that IADPSG criteria (25.4%), and ADA criteria (23.9%).

**Conclusions:**

Along with poor quality of reporting, publishing in non-indexed journals and significant disagreement between studies, the prevalence rate of GDM is high in Pakistan. Consensus building among stakeholders for recommended screening methods; and continuous medical education of the physicians are much needed for a timely detection and treatment of GDM.

## Introduction

Gestational diabetes mellitus (GDM) is considered as hyperglycemia during pregnancy among women without prior history of diabetes [1]. It can adversely affect the perinatal outcomes [2]; therefore, an early diagnosis and treatment is a key to improve the prognosis and reduce the adverse outcomes [3]. Universal screening for GDM does not seem cost effective in high-income countries. However, it might be valuable in low and middle income countries [4]. A 75g 2-h oral glucose tolerance test (OGTT) during 24 to 28 weeks is recommended as a gold standard method for the detection of GDM [5]. According to the International Association of Diabetes and Pregnancy Study Groups (IADPSG) [6], the World Health Organization (WHO) [7], and the American Diabetes Association (ADA) [5] criteria﻿, the cut-off values for the diagnosis of GDM are ≥ 92 mg/dl after fasting, ≥ 180 mg/dl after one-hour and ≥ 153 mg/dl after two-hour. A variety of other screening tools and criteria are used for the diagnosis of GDM.

In the last two decades, several research studies have been conducted in Pakistan that measured the prevalence of GDM or compared the diagnostic accuracy of GDM screening tools. These single-center hospital-based studies utilized one of the standard or non-standard screening methods and derived a prevalence rate varying from 4.41% to 57.90% [8–30]. Beside this disagreement, similar multi-centric studies, community surveys and pooled evidence were lacking from the country. Therefore, the current study aimed to measure the overall and subgroup pooled estimates of GDM and explore the methodological variations among studies for any inconsistency.

## Methods

### Protocol and registration

The study protocol was not prepared or registered. However, the review is done in accordance with Preferred Reporting Items for Systematic Reviews and Meta-Analyses (PRISMA) guidelines [31].

### Eligibility criteria

In consideration of the study outcome i.e. prevalence of GDM in Pakistan, the criteria for eligible studies were prospective observational studies, published as original research paper, in the English language, in any journal and on any date. Similarly, the criteria for eligible subjects were pregnant women of any age and gestational age, without any medical condition or history, from any socioeconomic class and any region of Pakistan.

### Information sources and search process

The first author searched in PubMed, ScienceDirect, Google Scholar and PakMediNet database for eligible records during the period from December 2022 to January 2023. In PubMed advanced search, using search field title/abstract and affiliation Pakistan, the keywords “gestational diabetes mellitus or GDM” and “oral glucose tolerance test or OGTT” were entered to find the eligible records. Then the search was further refined by using the filters as follows: observational study, specie human and language English. After that the filtered studies were ordered by “best match”; and their titles were assessed for relatedness. Similar searches were made on PakMediNet, Google Scholar and ScienceDirect databases.

### Screening of abstracts and full-text articles

**Fig. 1:**
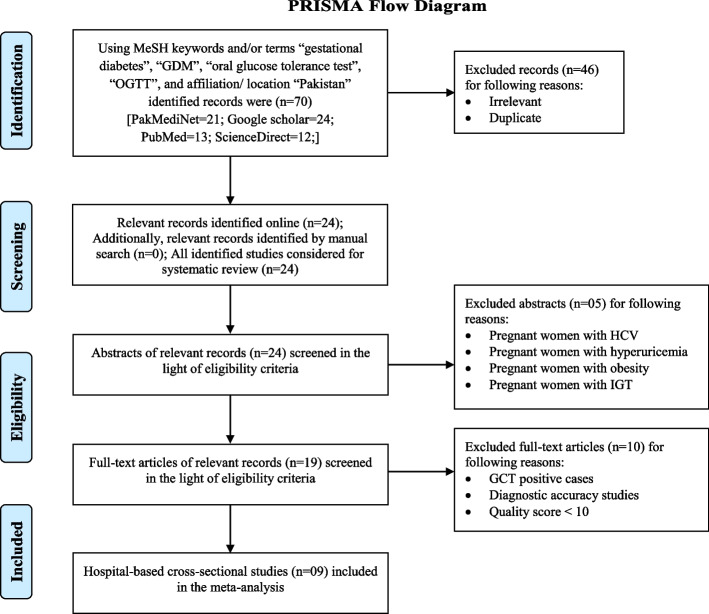
PRISMA flow diagram

After excluding irrelevant or duplicate records (*n* = 46) from identified records (*n* = 70), the relevant studies (*n* = 24) were considered for systematic review. However, their abstracts were screened and the studies (*n* = 05) that enrolled women with hepatitis C infection, hyperuricemia, obesity or IGT were excluded. Then, their full-text manuscripts (*n* = 19) were screened and the studies (*n* = 10) that measured GDM in GCT positive cases, or had a different objective such as diagnostic accuracy studies or could not achieve the AXIS quality score > 10 were excluded. As a result, nine observational, hospital-based studies were selected for the meta-analysis, see Fig. 1. With 100% agreement (Cohen kappa = 1), both authors agreed to include/ exclude the studies in this systematic review & meta-analysis.

### Quality assessment

The appraisal tool for cross-sectional studies (AXIS) [32] was adopted for measuring the quality and risk of bias within relevant studies (n = 24). The tool had twenty questions, and each question had three responses (yes, no & don’t know), increasing the score by one for each yes. Each study received a score between 0 and 20. Based on the AXIS score, the individual studies were categorized as good (> 15), fair (10—15) and poor (< 10). In addition, indexation of journals that published individual studies was evaluated for Web of Science, Scopus and Medline; and their recognition by HEC Pakistan.

### Data items and collection process

The data retrieved were as follows: last name of the first author, publication date, study design & setting, sample size, screening tool, GDM diagnostic criteria, prevalence of GDM, age & gestational age of the study population, AXIS score and indexation status of the journal. The first author retrieved and extracted the data and repeated the process several times for any inconsistency. The extracted data were neither combined nor transformed. None of the authors of selected articles was approached to seek the data.

### Data analysis

The data were entered using Microsoft Excel, and forest plots for overall and subgroup pooled estimates of GDM were created using the Meta-Analyst version 3.13 βeta [33]. Funnel plot for potential publication bias was created using the Comprehensive Meta-Analysis (CMA) [34]. The heterogeneity among studies and subgroups was measured using I^^2^ statistics [35]. Overall and subgroup pooled estimates of GDM were calculated using the random effects model [36].

## Results

### Selection process of eligible studies

Out of identified records (*n* = 70), the relevant studies (*n* = 24) were considered for systematic review. However, only 09 studies could meet the eligibility criteria, achieved AXIS quality score > 10 and selected for meta-analysis. The PRISMA flow diagram was used to indicate the methods for selecting studies, see Fig. 1.

### Characteristics of studies included in the meta-analysis

The year of publication of studies (*n* = 24) considered for systematic review ranged from 2002 to 2022; and of studies (*n* = 09) included in the meta-analysis ranged from 2015 to 2022. All included studies (*n* = 09) were prospective, observational, hospital-based studies published in the English language. The assessment of journals for indexation (*n* = 09) revealed that seven studies were published in Scopus indexed journals (two discontinued), four in WoS indexed journals, one in Medline indexed journal, six in HEC-Pak recognized journals, and two in non-indexed journals.

Six studies did not report inclusion criteria for age of study population. Four studies reported gestational age (24 to 28 weeks) of study population. Five studies reported adoption of 75g 2-h OGTT. The IADPSG and DIPSI criteria were adopted in two studies each, and the WHO, ADA, Carpenter and ACOG criteria in one study each. Noteworthy, five studies reported using the WHO, IADPSG or ADA criteria but only two of them actually adopted their cut-off values, see Table 1.

**Table 1:** Characteristics of studies included in the meta-analysis

Author	Year	City	Sample size	Age range (years)	Gestational age (weeks)	Screening tool	Diagnostic criteria	Cut-off values	Prevalence (%)	HEC-Pak recognized	AXIS score assigned/total	Weight assigned
Atta et al. [8]	2022	Jamshoro	12,450	NA	All trimesters	75 g 2-h OGTT	DIPSI	> 140	12.00	No	12/20	28.006
Latif et al. [9]	2022	Quetta	530	19 – 43	24 – 28	75 g 2-h OGTT	IADPSG	92(F),180(1 h), 153(2 h)	35.80	Yes	15/20	2.596
Gul et al. [10]	2021	Peshawar	126	NA	16 – 28	75 g 2-h OGTT	WHO	> 220	10.00	No	13/20	0.248
Faisal et al. [11]	2018	Rahim Yar Khan	160	15 – 44	Pre- & postpartum	NA	NA	NA	15.60	No	11/20	0.449
Khan et al. [12]	2018	Islamabad	380	22 – 45	16 – 36	NA	ADA	95(F),180(1 h), 155(2 h),140(3 h)	24.00	Yes	15/20	1.474
Riaz et al. [13]	2018	Karachi	11,430	NA	All trimesters	75 g 2-h OGTT	DIPSI	> 140	11.80	Yes	13/20	25.345
Ahmad et al. [14]	2018	Lahore	558	NA	24 – 28	50 g OGTT	ACOG	NA	8.42	Yes	11/20	0.917
Fatima et al. [15]	2017	Karachi	1210	NA	24 – 28	75 g 2-h OGTT	IADPSG	92(F),180(1 h), 153(2 h)	17.20	Yes	16/20	3.669
Bibi et al. [16]	2015	Peshawar	190	NA	24 – 28	NA	Carpenter	95(F),180(1 h), 155(2 h),140(3 h)	26.30	Yes	14/20	0.785

*NA* Not available, *OGTT* Oral glucose tolerance test, *DIPSI* Diabetes in Pregnancy Study Group India, *IADPSG* International Association of Diabetes and Pregnancy Study Groups consensus panel, *WHO* World Health Organization, *ADA* American Diabetes Association, *ACOG* American College of Obstetricians and Gynecologists

### Quality/ risk of bias

**Fig. 2:**
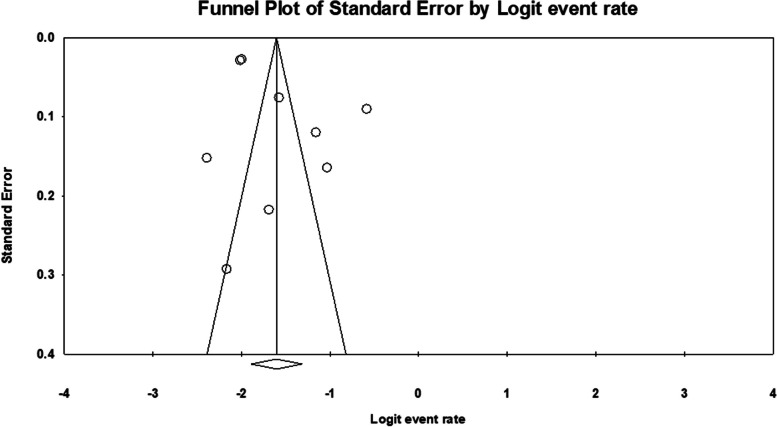
Funnel plot of standard error by logit event rate

All studies (*n* = 09) included in the meta-analysis were hospital-based, observational, with a clear objective of quantifying the burden of GDM in pregnant women without any comorbidity, and used OGTT as a screening tool. In addition, all of them achieved AXIS quality score > 10. Thus, the selection process minimized the heterogeneity among studies and subgroups to an insignificant level (I^^2^ < 50.0%). The publication bias was not seen in the funnel plot, see Fig. 2.

### Synthesis of results

**Fig. 3:**
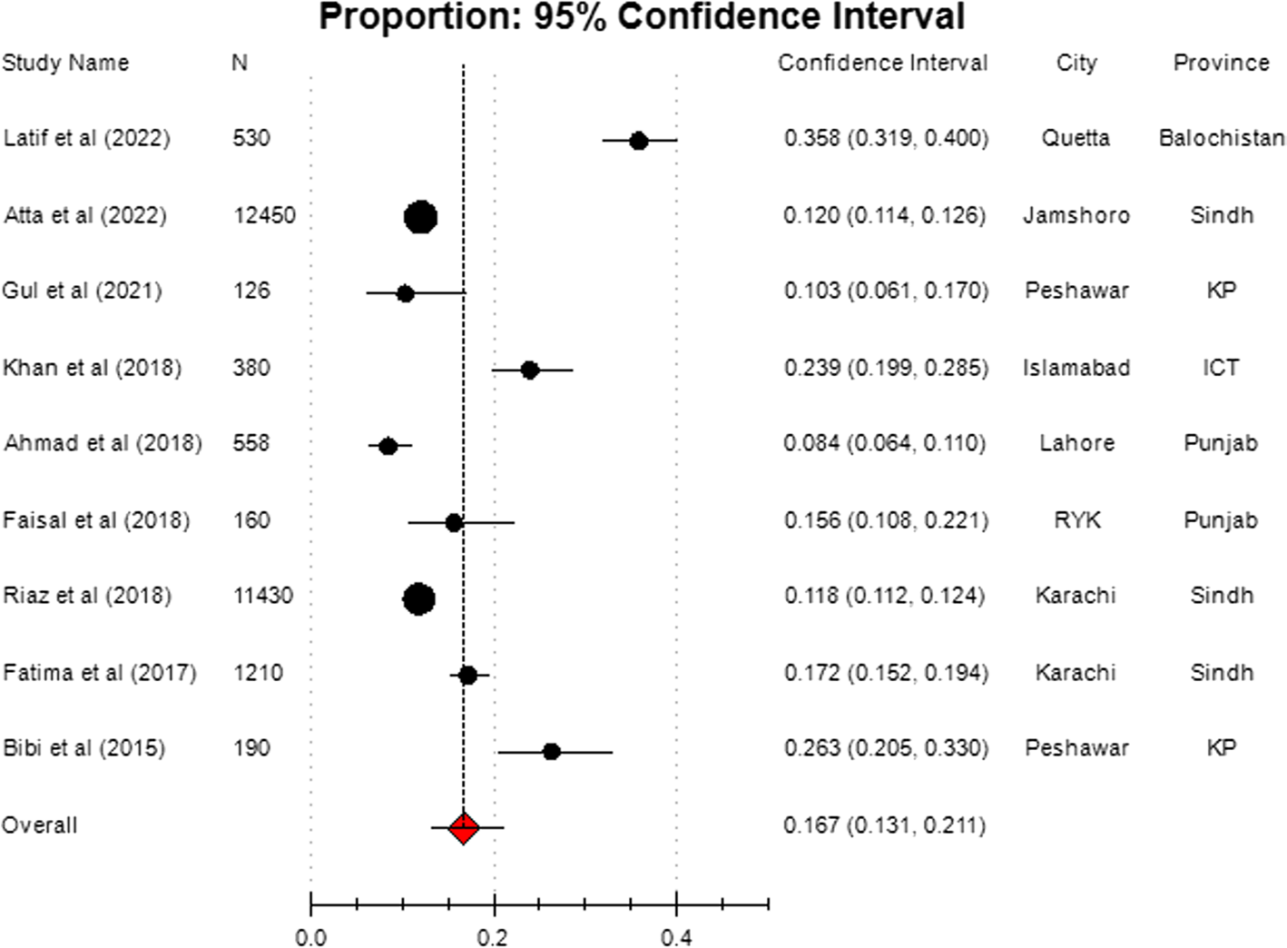
Forest plot for overall pooled estimate of GDM

The pooled sample of nine studies was 27,034 (126 – 12,450) pregnant women, of any gestational age, from different districts of all four provinces of Pakistan. The weight allocated to individual studies varied from 0.248 to 28.006. The prevalence of GDM among included studies ranged between 8.42% and 35.80%, see Table 1. The overall pooled estimate of GDM was 16.7% (95% CI 13.1 – 21.1), which was greater than GDM reported in five individual studies and less than of four studies, see Fig. 3.

### Subgroup analysis

**Fig. 4:**
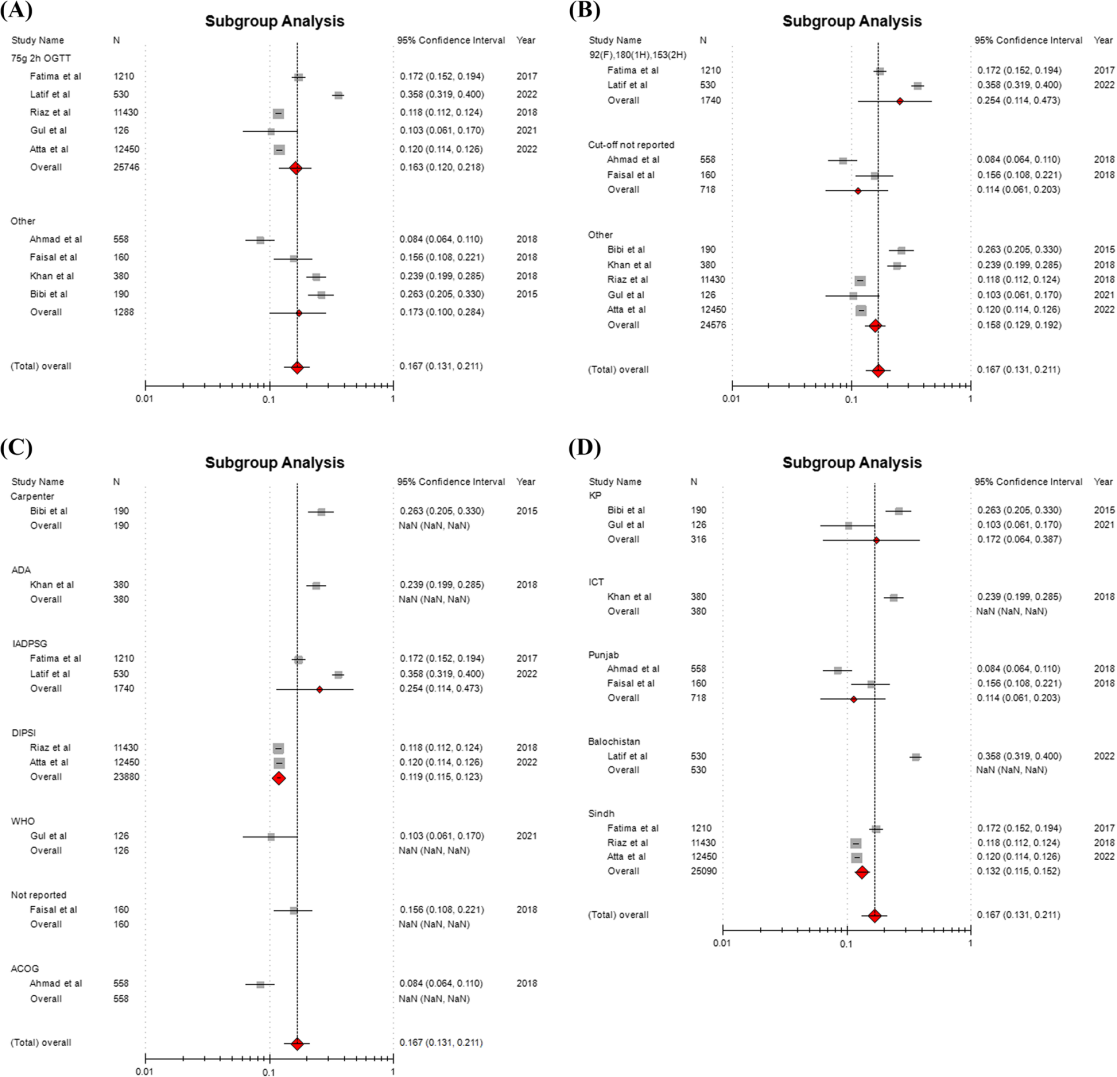
**A**-**D** Subgroup pooled estimates of GDM

The studies (*n* = 5) that adopted 75g 2-h OGTT had a little lower subgroup pooled estimate of GDM as compared to the studies (*n* = 4) that did not adopt or report standard OGTT (16.3% vs. 17.3%), see Fig. 4A. The studies (*n* = 2) that adopted diagnostic cut-off values [≥ 92 (F), ≥ 180 (1-h) and ≥ 153 (2-h)] by IADPSG, ADA and WHO had a higher subgroup pooled estimate of GDM as compared to the studies (*n* = 7) that adopted other or did not report diagnostic cut-off values (25.4% vs. 15.8%), see Fig. 4B. The studies that adopted Carpenter criteria demonstrated the highest subgroup pooled estimate of GDM (26.3%), after that IADPSG criteria (25.4%), and ADA criteria (23.9%), see Fig. 4C. The studies from Balochistan demonstrated the highest subgroup pooled estimate of GDM (35.8%), after that ICT Islamabad (23.9%), Khyber Pakhtunkhwa (17.2%), Sindh (13.2%), and Punjab (11.4%), see Fig. 4D.

### GDM in GCT positive cases

There were four studies that first utilized GCT for screening purposes and then performed 75g 2-h or 100g 3-h OGTT to diagnose GDM in GCT positive cases. Only two of them were published in HEC-Pak recognized journals and none of them could achieve AXIS score > 10. Among these studies, the prevalence of GDM ranged from 7.69% to 17.39%. Other characteristics are shown in Table 2.

**Table 2:** GDM in GCT positive cases

Author	Year	City	Sample size	GCT Positive %	GCT Positive cases	Age range (years)	Gestational age (weeks)	Screening tool	Diagnostic criteria	Cut-off values	Prevalence (%)	HEC-Pak recognized	AXIS score assigned/total
Sohag et al. [17]	2013	Karachi	585	40.10	235	32—38	> 24	100 g 3-h OGTT	NA	105(F),190(1 h), 165(2 h),145(3 h)	16.17	No	08/20
Jaleel et al. [18]	2009	Karachi	100	23.00	23	NA	NA	75 g 2-h OGTT	WHO	NA	17.39	No	09/20
Hassan et al. [19]	2005	Peshawar	1000	22.60	226	21—42	24—36	100 g 3-h OGTT	NA	NA	16.37	Yes	06/20
Javaid et al. [20]	2002	Lahore	1000	10.40	104	NA	24—28	NA	NA	NA	7.69	Yes	08/20

*NA* Not available, *OGTT* Oral glucose tolerance test, *WHO* World Health Organization

### GDM in women with comorbidity

There were five studies that diagnosed GDM in pregnant women having a medical condition such as hepatitis C infection, obesity, hyperuricemia or IGT. Only three of them were published in HEC-Pak recognized journals and two of them could achieve AXIS score > 10. Among these studies, surprisingly higher prevalence of GDM was observed in cases with hyperuricemia (57.90%), followed by hepatitis C (35.53%) and obese (23.70%). Other characteristics are shown in Table 3.

**Table 3:** GDM in women with comorbidity

Author	Year	City	Sample size	Comorbidity	Age range (years)	Gestational age (weeks)	Screening tool	Diagnostic criteria	Cut-off values	Prevalence (%)	HEC-Pak recognized	AXIS score assigned/total
Qamar et al. [21]	2022	Karachi	197	Hepatitis C	16—45	24—28	75 g 2-h OGTT	ADA	92(F),180(1 h), 153(2 h)	35.53	Yes	10/20
Hafeez et al. [22]	2020	Multan	190	Obesity	20—40	20—28	NA	NA	NA	23.70	No	09/20
Sahibzada et al. [23]	2020	Peshawar	187	Obesity	15—45	NA	2-h OGTT	NA	100(F),140(R)	22.00	No	12/20
Ismail et al. [24]	2019	Rawalpindi	140	Hyperuricemia	20—40	> 24	75 g 2-h OGTT	NA	92(F),180(1 h), 153(2 h)	57.90	Yes	10/20
Ali et al. [25]	2018	Peshawar	200	IGT	16—40	≤ 28	2-h OGTT	NA	NA	10.00	Yes	11/20

*NA* Not available, *OGTT* Oral glucose tolerance test, *IGT* Impaired glucose tolerance, *ADA* American Diabetes Association

### GDM in Diagnostic accuracy studies

There were five studies that compared the diagnostic accuracy of different screening tools including GCT, OGTT (DIPSI) or HbA1c with a 75g 2-h OGTT or 100g 3-h OGTT taking as the gold standard method for diagnosing GDM. Though all of them were published in HEC-Pak recognized journals; however, none of them could achieve AXIS score > 10. Among these studies, the prevalence of GDM ranged from 4.41% to 27.71% by OGTT (tool 1); and from 12.80% to 31.46% by other methods (tool 2). Other characteristics are shown in Table 4.

**Table 4:** GDM in diagnostic accuracy studies

Author	Year	City	Sample size	Age range (years)	Gestational age (weeks)	Screening Tool 1	Diagnostic criteria	Cut-off values	Prevalence (%) 1	Screening Tool 2	Prevalence (%) 2	HEC-Pak recognized	AXIS score assigned/total
Ismail et al. [26]	2022	Karachi	351	NA	24—32	75 g 2-h OGTT	WHO	> 140	11.68	2-h OGTT DIPSI	12.80	Yes	10/20
Masood et al. [27]	2021	Karachi	204	NA	All trimesters	75 g 2-h OGTT	WHO	126(F),140(R)	4.41	2-h OGTT DIPSI	15.68	Yes	10/20
Arif et al. [28]	2021	Lahore	100	20—35	24—28	100 g 3-h OGTT	Carpenter	95(F),180(1 h), 155(2 h),140(3 h)	10.00	GCT	19.00	Yes	05/20
Khan et al. [29]	2020	Islamabad	280	NA	16—32	75 g 2-h OGTT	IADPSG	NA	19.28	HbA1c	17.85	Yes	10/20
Fatima et al. [30]	2018	Faisalabad	267	15—39	24—28	75 g 2-h OGTT	NA	126(F),200(R)	27.71	GCT	31.46	Yes	07/20

*NA* Not available, *OGTT* Oral glucose tolerance test, *WHO* World Health Organizationm, *IADPSG* International Association of Diabetes and Pregnancy Study Groups consensus panel, *DIPSI* Diabetes in Pregnancy Study Group India, *GCT* Glucose challenge test, *HbA1c* Glycosylated hemoglobin

## Discussion

GDM can adversely affect the pregnancy related maternal and neonatal outcomes [2]; therefore, pregnant women undergo screening for GDM during pregnancy. A variety of screening tools and criteria are used for the diagnosis of GDM. As a result, the prevalence rate of GDM varied from 4.41% to 57.90% among studies from Pakistan [8–30]. Beside this disagreement, similar multi-centric studies, community surveys and pooled evidence were lacking from the country. Therefore, this meta-analysis measured the overall pooled estimate of GDM, which was 16.7% (95% CI 13.1 – 21.1). In a meta-analysis measuring GDM prevalence in Eastern Mediterranean region, Badakhsh et al. included four studies from Pakistan and reported an equivalent subgroup pooled estimate of GDM 15.3% (95% CI 9.4 – 21.2) [37]. However, in another meta-analysis measuring GDM prevalence in Asia, Lee et al. selected two studies from Pakistan and reported a pooled prevalence 7.7% (95% CI 6.4 – 9.0) [38] that was less than half of the GDM estimated in current study. This review also revealed that the highest prevalence of GDM 57.90% was observed in pregnant women with hyperuricemia [24], which is regarded as a novel and significant risk factor of GDM [39]. Similarly, a higher prevalence of GDM 35.53% in HCV positive women [21], and ≥ 22.0% in obese women [22, 23] demonstrated that HCV infection [40] and obesity [41] had a greater risk of developing GDM. These findings suggested that pregnant women with a comorbid condition should get more attention for having higher risk of GDM.

Indexation of a journal is taken as a measure of its quality and indexed journals are regarded as having more scientific quality than non-indexed journals [42]. However, it is of a serious concern that majority of the studies included in this review could not achieve a good (> 15) quality score and were published in the non-indexed journals. Several studies did not even mention institutional ethical approval, sample size justification, sampling method, diagnostic criteria, cut-off values and/ or study limitations. Surprisingly, none of them talked about non-response rate, non-response bias and non-respondent characteristics [8–30].

It is evident from the present study that there was great variation across studies in terms of study population, study design, sample selection, screening tool and diagnostic criteria. Similar concerns had been raised in other studies from Pakistan. In a nationwide cross-sectional survey, Askari et al. indicated major differences for screening, diagnosis and management of GDM among physicians, gynecologists and endocrinologists. Where 52.9% of them were practicing universal screening and 47.1% were in favor of selective screening of GDM. For the diagnosis of GDM, 40.4% of them were using estimation of plasma glucose fasting/ random, followed by 75g OGTT (25.1%), 50g GCT (20.0%), urine glucose (8.0%), HbA1c (5.2%) and 100g OGTT (1.3%) [43]. Similar results were reported by Riaz et al., where 51.43% healthcare professionals were using estimation of plasma glucose fasting (26.19%) & random (25.24%) for the diagnosis of GDM, followed by 75g OGTT (24.29%), hemoglobin A1c (9.52%), and 50g OGTT (4.29%). In addition, the authors observed lack of agreement for screening methods and management of GDM and suggested that the doctors need to be educated to adopt standard diagnostic methods and management guidelines [44].

## Conclusions

Along with poor quality of reporting, publishing in non-indexed journals and significant disagreement between studies, the prevalence rate of GDM is high in Pakistan. Consensus building among stakeholders for recommended screening methods; and continuous medical education of the physicians are much needed for a timely detection and treatment of GDM.

### Strengths and limitations

To the best of our knowledge, it is the first systematic review and meta-analysis on the topic from Pakistan. The purposive inclusion of studies having AXIS score > 10 minimized the heterogeneity among studies and subgroups to an insignificant level (I^^2^ < 50.0%). The study protocol was not prepared or registered. The first author retrieved and extracted the data and repeated the process several times for any inconsistency. The extracted data were neither combined nor transformed. None of the authors of selected articles was approached to seek the data.

## Data Availability

The datasets used and/or analyzed during the current study are available from the corresponding author on reasonable request.
